# Concordance between radioimmunoassay and fixed cell‐based assay in subjects without myasthenia gravis: optimizing the diagnostic approach

**DOI:** 10.1111/ene.16435

**Published:** 2024-08-08

**Authors:** Silvia Falso, Sofia Marini, Cinzia Carrozza, Eleonora Sabatelli, Giovanni Mascagna, Martina Marini, Jacopo Morroni, Amelia Evoli, Raffaele Iorio

**Affiliations:** ^1^ Department of Neuroscience Università Cattolica del Sacro Cuore Rome Italy; ^2^ Department of Chemistry and Clinical Biochemistry Fondazione Policlinico Universitario Agostino Gemelli IRCCS Rome Italy; ^3^ Neurology Unit Fondazione Policlinico Universitario Agostino Gemelli IRCCS Rome Italy

**Keywords:** autoantibodies, autoimmune, diagnostic tests, nervous system, neuromuscular junction

## Abstract

**Background and Purpose:**

Acetylcholine receptor antibody (AChR‐Ab) detection is crucial in myasthenia gravis (MG) diagnosis and, currently, the radioimmunoassay (RIA) is the gold standard. However, RIA may detect AChR‐Ab against nonpathogenic intracellular epitopes. In this study, we performed fixed cell‐based assay (F‐CBA) in RIA‐AChR‐Ab positive subjects without MG symptoms, to assess whether F‐CBA could show a higher specificity compared to RIA in detecting pathogenic Abs.

**Methods:**

We reviewed medical records of patients referred to our MG outpatient clinic because of RIA‐AChR‐Ab detection. MG diagnosis was based on clinical examination, electrophysiology and Ab detection. AChR‐Abs were tested by RIA in the whole cohort. Serum samples from RIA‐positive asymptomatic subjects were retested by F‐CBA.

**Results:**

Of 605 subjects who tested RIA‐AChR‐Ab positive, MG diagnosis was confirmed in 599. Six subjects were RIA‐AChR‐Ab positive although they had never had MG symptoms; in four of these subjects AChR‐Abs were not detected by F‐CBA, whereas the remaining two (both non‐MG thymoma cases) were positive also by F‐CBA.

**Conclusions:**

RIA false positivity for AChR‐Ab is very rare. Previous literature has demonstrated that F‐CBA has higher sensitivity than RIA for MG, especially in ocular cases. Our preliminary results show that, in rare instances, F‐CBA may be more specific than RIA for MG diagnosis.

## INTRODUCTION

Myasthenia gravis (MG) is an autoimmune disease caused by autoantibodies (Abs) binding to the extracellular domain of antigens expressed at the motor endplate and resulting in muscle fatigability [1]. Ab detection is crucial for MG diagnosis. Overall, Abs against the nicotinic acetylcholine receptor (AChR) are detected in approximately 85% of patients with generalized symptoms and in up to 65% with pure ocular MG [2]. Most pathogenic anti‐AChR Abs target the extracellular domain of the α1 subunit, where the main immunogenic region is located [3]. Up to 40% of AChR‐negative patients harbor Abs against muscle‐specific kinase (MuSK), and 10%–15% of patients have no detectable Abs by radioimmunoassay (RIA) and are considered double seronegative. For the past decade, high‐sensitive live cell‐based assay (L‐CBA), using human embryonic kidney 293 cells transfected to express clustered AChR isoforms or MuSK on the cell surface, has allowed the detection of “clustered AChR antibodies” in 16%–50% of the seronegative population [4, 5, 6, 7, 8]. More recently, a commercial fixed CBA (F‐CBA) for adult and fetal AChR and MuSK Ab detection has become available and, like L‐CBA, has proven to be highly sensitive and specific for MG diagnosis [9].

With the advent of new therapies for MG, precise diagnosis and dependable antibody testing become foundational to effective treatment strategies [10]. The aim of this study was to assess the performance of F‐CBA in RIA‐AChR‐Ab positive subjects with no symptoms of MG, in order to evaluate the F‐CBA capacity to detect Abs with clinical relevance.

## METHODS

### Study population

We reviewed medical records of patients referred to the MG outpatient clinic of Fondazione Policlinico Universitario Agostino Gemelli IRCCS because of AChR‐Ab detection by RIA between 1 January 2000 and 31 January 2022. Positive subjects who performed the diagnostic test for reasons other than MG symptoms were retrospectively identified. Clinical follow‐up was ≥2 years.

The AChR‐MG diagnosis was based on the typical clinical pattern of fluctuating muscle weakness that improved after rest, coupled with evidence of neuromuscular transmission impairment on electrophysiological studies. This included a decrement of the compound muscle action potential of >10% during low‐rate repetitive nerve stimulation and/or increased jitter on single‐fiber electromyography (SF‐EMG).

### Ab detection

The entire cohort of patients was initially tested by RIA, following the manufacturer's instructions (RSR Limited, Cardiff, UK) with values > 0.5 nmol/L considered positive.

Based on the inclusion criteria, we retrospectively identified six RIA‐AChR‐Ab positive subjects without MG symptoms for enrollment in this study. These subjects were invited to new clinical examination and Ab testing. All patients accepted and, at the time of the visit, blood samples were collected and stored at −80°C until analysis. Samples were concurrently tested by F‐CBA and RIA. RIA was performed exclusively in the laboratory of Fondazione Policlinico Universitario Agostino Gemelli IRCCS, Rome (RSR Limited). Serum samples were tested for Abs to the adult/fetal AChR with commercial F‐CBA (Euroimmun, Lubeck, Germany), performed according to the manufacturer's instructions (serum dilution 1:10). F‐CBA results were evaluated using a fluorescence microscope by two raters blind to previous results. The interrater agreement was 100%.

### Ethical standards

The study was approved by the institutional review boards of Fondazione Policlinico Universitario Agostino Gemelli IRCCS (protocol #23752/14). All patients involved gave informed consent to the use of their medical records for research purposes. The study was performed in accordance with the ethical standards laid down in the 1964 Declaration of Helsinki and its later amendments.

## RESULTS

Between 1 January 2000, and 31 May 2022, our tertiary center consecutively evaluated 605 subjects who tested positive for AChR‐Abs using RIA. In 599 of these subjects, the diagnosis of MG was further confirmed based on clinical features and electrodiagnostic studies. However, in six subjects (1%), AChR‐Abs detected by RIA had never been associated with symptoms of MG. Two of these subjects had undergone thymectomy for B2 thymoma, with postoperative follow‐up of 14 and 13 years, respectively, and had consistently tested positive for AChR‐Abs by RIA since their diagnosis of thymoma. Both patients also underwent SF‐EMG with negative results. At the time of this study, their RIA values were 6.30 nmol/L and 6.80 nmol/L, respectively. Furthermore, the F‐CBA clearly indicated positivity for both fetal and adult AChR‐Abs in these patients.

**FIGURE 1:**
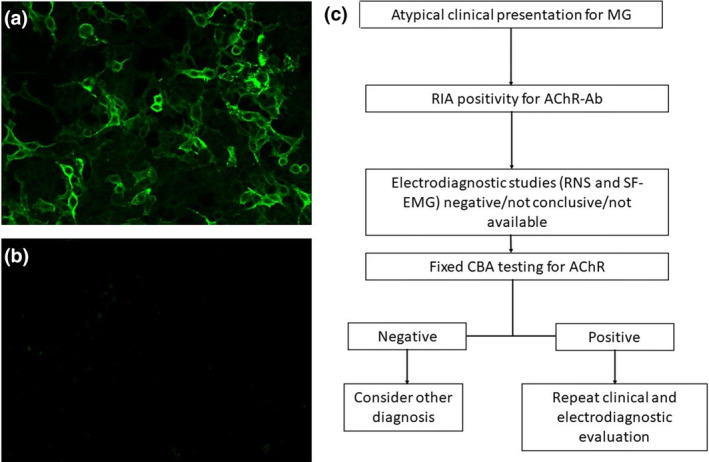
(a, b) Fixed cell‐based assay (CBA) demonstrates a positive result for adult acetylcholine receptor (AChR) autoantibodies (Abs) when tested with serum from a patient with myasthenia gravis (MG; a), contrasting with the lack of immunoreactivity observed in serum from a patient without typical symptoms of MG, despite testing positive for AChR‐Abs by radioimmunoassay (RIA; b). (c) Flowchart showing the diagnostic approach when MG is deemed atypical on clinical grounds. RNS, repetitive nerve stimulation; SF‐EMG, single‐fiber electromyography.

In four other subjects, AChR‐Ab testing was conducted without prior clinical screening (refer to Table 1). The RIA‐positivity for AChR‐Abs (5.07, 4.80, 2.31, and 2.30 nmol/L) led to a neurological examination and electrophysiological testing. In two of these cases, chest computed tomography scans revealed thymic hyperplasia; one of these cases received pathological confirmation of the condition following thymectomy. Electrophysiological studies in all four cases did not demonstrate neuromuscular transmission impairment (detailed in Table 1). The initial RIA positivity for AChR‐Abs was not corroborated by F‐CBA results (Figure 1b). Notably, none of the six subjects had received treatment with steroids or immunosuppressive drugs. Detailed clinical and laboratory features of these patients are provided in Table 1.

**TABLE 1:** Demographic, clinical, and electrodiagnostic characteristics of RIA‐AChR‐Ab positive non‐MG subjects.

Patient/sex/age, years	RIA, nmol/L	F‐CBA	Reason to perform RIA	Thymus	RNS	SF‐EMG	Treatment	Follow‐up, years
1/F/52	6.80	Positive	Evidence of thymoma at chest CT scan performed for asthma	Thymoma B2	Negative	Negative	No	14
2/M/45	6.30	Positive	Evidence of thymoma at chest MRI performed for myocarditis	Thymoma B2	Negative	Negative	No	13
3/F/55	5.07	Negative	Evidence of thymic hyperplasia at chest CT scan performed for tachycardia	Thymic hyperplasia	Negative	Negative	No	3
4/M/62	4.80	Negative	Low back pain	N/A	Negative	N/A	No	2
5/F/55	2.31	Negative	Autoimmune screening	Normal	Negative	Negative	No	9
6/F/38	2.30	Negative	Widespread pain and cramps	Thymic hyperplasia	Negative	N/A	No	2

Abbreviations: Ab, antibodies; AChR, acetylcholine receptor; CT, computed tomography; F‐CBA, fixed cell‐based assay; MG, myasthenia gravis; MRI, magnetic resonance imaging; N/A, not available; RIA, radioimmunoassay; RNS, repetitive nerve stimulation; SF‐EMG, single‐fiber electromyography.

## DISCUSSION

RIA is considered very specific for MG diagnosis, although it can also detect nonpathogenic AChR‐Abs against intracellular epitopes and can be rarely positive in other neurological diseases. Maddison et al. reported five RIA‐AChR‐Ab positive patients with diagnoses other than MG who were found to be negative by L‐CBA, suggesting false‐positive results from the initial RIA [11]. CBA is deemed to be more sensitive than RIA in confirming MG diagnosis, especially in ocular cases, as it can detect low‐affinity Abs [4, 5, 6, 7, 8]. Our study results, although preliminary, show that F‐CBA may also be more specific than RIA in detecting pathogenic Abs.

F‐CBA was positive in two thymoma patients without MG, suggesting that these subjects had Abs against AChR without clinical symptoms or neurophysiological signs of MG (for 14 and 13 years), as already described [12]. One may speculate that these Abs are not pathogenic despite targeting AChR. In the four F‐CBA‐negative patients, MG was further excluded by negative electrodiagnostic studies. In addition, AChR titers were >2.30 nmol/L in the whole cohort (see Table 1). Altogether, these data are against the hypothesis that these samples were F‐CBA “false negative” and suggest that AChR‐Abs detected by RIA were not pathogenic or specific for intracellular epitopes of AChR. Likewise, the evidence that contrast voltage‐gated potassium channel Abs detected by RIA target intracellular epitopes without pathogenic potential, and that Abs to leucine‐rich glioma‐inactivated 1 (LGI1) and contactin‐associated protein like‐2 (CASPR2) target surface‐exposed domains of LGI1 or CASPR2, being directly pathogenic, has driven the need for CBA use in clinical practice [13].

Recently, a multicenter, prospective, double‐blind study compared the accuracy of diagnostic assays in myasthenia gravis. F‐CBA showed 11.3% increased sensitivity with 97.8% specificity in AChR‐Ab detection compared with RIA (cutoff for positivity = 0.5 nmol/L) [14]. Mirian et al. [15] found 4% increased sensitivity with 99% specificity for F‐CBA versus RIA. Our study underlines the importance of testing for AChR‐Abs only when clinical features suggestive of MG are detected. However, in real‐world clinical practice, diagnostic tests are not always requested by MG experts. Our findings suggest that F‐CBA may be performed as an additional tool to increase diagnostic accuracy when clinical presentation and electrodiagnostic studies are deemed atypical for MG. Of late, improved recognition of MG has facilitated earlier diagnosis and contributed to an apparent increase in disease prevalence. However, this also entails the risk of MG overdiagnosis.

In conclusion, our findings indicate that AChR detection by F‐CBA may offer greater specificity for identifying pathogenic AChR‐Abs compared to RIA. Future research should investigate whether these likely nonpathogenic Abs bind to intracellular epitopes of the AChR or to other antigens complexed with the receptor.

## AUTHOR CONTRIBUTIONS

**Silvia Falso:** Conceptualization; investigation; data curation; formal analysis; writing – original draft; visualization; validation. **Sofia Marini:** Investigation; validation; visualization. **Cinzia Carrozza:** Methodology; investigation; validation. **Eleonora Sabatelli:** Investigation; validation; visualization. **Giovanni Mascagna:** Methodology; investigation; validation. **Martina Marini:** Investigation; validation; visualization. **Jacopo Morroni:** Investigation; validation; visualization; writing – review and editing. **Amelia Evoli:** Data curation; investigation; validation; writing – review and editing. **Raffaele Iorio:** Conceptualization; data curation; investigation; validation; funding acquisition; visualization; resources; supervision; project administration; software; writing – review and editing; methodology; formal analysis.

## CONFLICT OF INTEREST STATEMENT

R.I. has received consultancy fees and speaker honoraria from Alexion, Argenx, UCB, and Dianthus Therapeutics. None of the other authors has any conflict of interest to disclose.

## Data Availability

The data that support the findings of this study are available from the corresponding author upon reasonable request.
